# MRI analysis of the physiological patellofemoral joint morphology of adult knees

**DOI:** 10.1007/s00256-024-04794-1

**Published:** 2024-09-24

**Authors:** Marc-Pascal Meier, Yara Hochrein, Mark-Tilmann Seitz, Paul Jonathan Roch, Katharina Jäckle, Ali Seif Amir Hosseini, Wolfgang Lehmann, Thelonius Hawellek

**Affiliations:** 1 Department of Trauma Surgery, Orthopaedics and Plastic Surgery, University Medical Center Goettingen, Robert-Koch-Straße 40, 37075 Göttingen, Germany; 2 Department of Diagnostic and Interventional Radiology, University Medical Center Goettingen, Robert-Koch-Straße 40, 37075 Göttingen, Germany

**Keywords:** Patellofemoral morphology, Patellofemoral joint, MRI, Knee morphology, Knee arthroplasty

## Abstract

**Objective:**

The aim of the present study was to determine physiological reference values for the morphology of the patella and to analyse these parameters according to patella position in healthy knee joints.

**Material and methods:**

Healthy knee joints of 409 patients (mean age, 52.3 years [± 16.8]) were analysed retrospectively on MRI images for Insall-Salvati index (ISI), sagittal patella thickness (PTS) and patella length (PLS) as well as axial patella thickness (PTA) and patella width (PWA). Differences between patellar diameters were analysed depending on ISI, side, age and gender.

**Results:**

Mean PTS was 20.1 mm (± 2.4), PLS 44.0 mm (± 4.4), PTA 21.8 mm (± 2.4) and PWA 44.5 mm (± 4.7). Depending on the vertical patellar position (ISI), all patellar parameters (*p* < 0.01) showed significant differences between patients with a patella alta, norma and baja. In general, a smaller ISI showed higher measured values for the patellar parameters. There were no significant differences for the laterality. Only PTS showed a significant age difference (*p* = 0.031). All parameters were significantly larger in male compared to female knees (*p* < 0.001).

**Conclusion:**

Reference parameters for the patella morphology are reported. Concluding from the results, a relationship between vertical patellar position and patellar morphology seems to exist. This finding should be taken into account in diagnostics and therapy of patella disorders.

## Introduction

The patella in its function as a sesamoid bone is of great biomechanical importance for the knee joint, as it transmits the muscle pull of the quadriceps femoris to the lower leg and thus enables active knee joint extension [1–3]. Both a low patellar (patella baja) as well as a high patellar position (patella alta) can have a negative impact on the biomechanics of the patellofemoral joint and cause pain symptoms [4, 5]. Anterior knee pain (AKP) is a symptom that occurs in both young athletes and older patients as part of patellar disorders [6–9]. Often clearly diagnosable etiologies such as malalignment, dysplasia and structural or implant-associated damage could be found in further diagnostics. However, there is a relevant number of cases in which subsequent examinations do not reveal any pathological condition [10–13]. The etiology of AKP often remains uncertain if no radiological pathologies can be found [10, 14–16]. The patellar height position could be a reason for joint instability and could influence the occurrence of AKP [17–20]. There are different parameters for determining the patella height position. According to current studies, the Insall-Salvati index (ISI) has the highest quality in an intermodal comparison between X-ray imaging and magnetic resonance tomography. It also shows good reliability [21]. Correlations between the ISI and the patellar morphology, in particular the patellar diameters, have not yet been sufficiently researched.

In everyday clinical practice, physiological radiological reference values for the morphology of the patellofemoral joint are required to enable the best possible diagnostics of uncertain knee pain. However, only a few studies focus on an accurate assessment of the physiological morphology of the patellofemoral joint [20–24]. The current literature provides evidence for gender-specific differences in patellofemoral morphology and the accuracy of the currently available physiological reference values for vertical patellar height position is controversial [21, 25–27]. Furthermore, dependencies between the patellar parameters and the patellar height position have not been sufficiently analysed.

The aim of the present study was therefore to determine physiological reference parameters for patellar morphology in healthy knee joints, and moreover to examine these parameters with the corresponding patellar height position. In addition, the patellar diameters should be examined with respect to side, age and gender. The results of the present study should serve as a radiological reference for the physiological morphology of patellofemoral joint and contribute to improve the diagnostics and therapy of AKP in preserving and replacing knee joint surgery.

## Materials and methods

### Patients

Between 2007 and 2020, a total of *n* = 5 627 patients underwent a magnetic resonance imaging of the knee joint in the Department for Diagnostic and Interventional Radiology of University Medical Center Goettingen. These MRIs were reviewed retrospectively. After applying inclusion and exclusion criteria, 409 patients were included in the final analysis. The study was approved by the local ethics committee (IRB number 35/7/20) and performed in accordance with the principles expressed in the Declaration of Helsinki. Without exception, the evaluated MRI were taken as part of routine diagnostics because of clinical symptoms. All MRIs were assessed by a senior radiologist and YH, MPM, ASAH and TH to exclude extended structural injuries or significant joint degeneration.

### Inclusion criteria

All examinations accessible via PACS system (Picture Archiving and Communication System) between January 1, 2007 and December 31, 2020 were initially included in the study. Out of these, all patients with an age of 20 years or more were included. All MRI scans were performed on patients to assess knee joint pathologies. They were examined by the internal radiology department as part of the clinical diagnostic procedure. Every report was re-evaluated by YH, MPM, ASAH and TH in a blinded fashion.

### Exclusion criteria

All patients with a Kellgren/Lawrence score (KL) [28] > 2, fractures, osteonecrosis, dysplasia, ligamentary damage or tumors were excluded. Patients who had undergone osteosynthesis or arthroplasty were likewise excluded. Similarly, the data did not include patients who had any other implants after knee joint preservation surgery. Low-quality MRIs (based on only a few gates) were excluded. In addition, all MRIs with imaging artefacts were ruled out.

### MRI analysis, parameters and methods of measurement

**Fig. 1:**
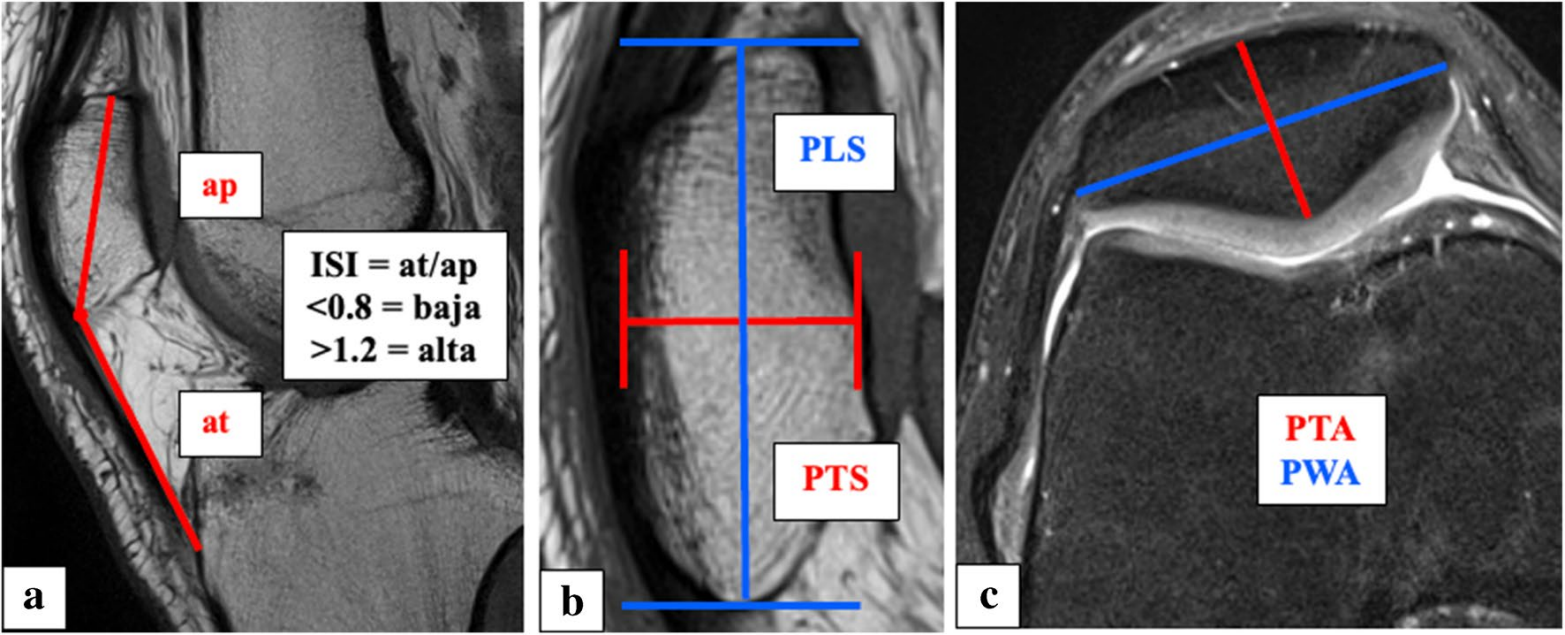
Exemplary depiction of the determination of Insall-Salvati-index (ISI, **a**), thickness of patella in sagittal view and length of patella in sagittal view (PTS and PLS, **b**) and thickness of patella in axial view and wide of patella in axial view (PTA and PWA, **c**). The parameter “ap” defines the greatest pole-to-pole length of patella, while “at” indicates the length of the posterior surface of the tendon from the lower pole of the patella to its insertion on the tibia. The result of dividing “at” by “ap” provides the ISI (**a**). PTS and PLS were measured in the sagittal view. The plane with the maximum extension of surface of the patella was selected (**b**). Likewise, PTA and PWA were measured in the axial plane (**c**)

All measurements were taken via the PACS system (Picture Archiving and Communication System). Software from GE Healthcare called Centricity™ Universal Viewer was used (RA1000, edition 2019, Buckinghamshire, Great Britain). The osteoarthritis score of each knee joint was classified according to Kellgren/Lawrence [28]. The thickness of patella was captured in sagittal and axial views (PTS, PTA). Moreover, the length of patella in a sagittal view (PLS) and width of patella in an axial view (PWA) were measured. For the determination of ISI, the greatest pole-to-pole length of patella (ap) and the length of the posterior surface of the tendon from the lower pole of the patella to its insertion on the tibia (at) were recorded. The result of dividing “at” by “ap” provides the ISI. All parameters were measured with established methods [19, 22, 29, 30]. Figure 1 shows the measurement methodology in principle. All radiographic parameters in this MRI study were manually measured separately in a standardized manner by the same observer (YH) under supervision of MPM, ASAH and TH. Intra-observer reliability of the measurements of all parameters was assessed for a subset of 50 subjects by blinded re-evaluation at 2 weeks after the first measurement and using the same technique. Inter-observer reliability was assessed by two observers (YH and MPM) independently for 50 subjects.

### Statistics

The analysis of side-, age- and gender-specific differences of PTS, PLS, PTA, PWA was based on Mann–Whitney *U* test. Patients were divided into age groups above and below 50 years. Ordinary one-way ANOVA was performed for comparison of patellar diameters in dependency of ISI. According to the ISI, the collective was divided up in patella alta, norma and baja. The Tukey’s multiple comparison test between alta/norma, alta/baja and norma/baja was used to accurately record single group differences. Intra- and inter-observer reliabilities were evaluated using intraclass correlation coefficients (ICC). Overall, mean ± standard deviation was stated. Minimum and maximum were determined for patellar diameters. Statistical analysis was performed with GraphPad Prism 9.00 (GraphPad Software, San Diego, USA), SPSS Statistics software version 27.0 (IBM SPSS Inc., Chicago, IL, USA) and Microsoft Excel (Microsoft Office 2016, Redmond, USA). Significant differences are marked with asterisks (***p < 0.001, **p < 0.01, *p < 0.05).

## Results

### Characteristics of the study population

In this study, 198 male (48.4%) and 211 female (51.6%) knee joints without radiological structural damage or a Kellgren/Lawrence score of 0–2 were analysed. Likewise, 197 right (48.2%) and 212 left (51.8%) knees were included in the final analysis. The mean age of the study population was 52.3 years (± 16.8 [age range, 21–88 years]).

### Analysis of intra-observer and inter-observer reliability

**Fig. 2:**
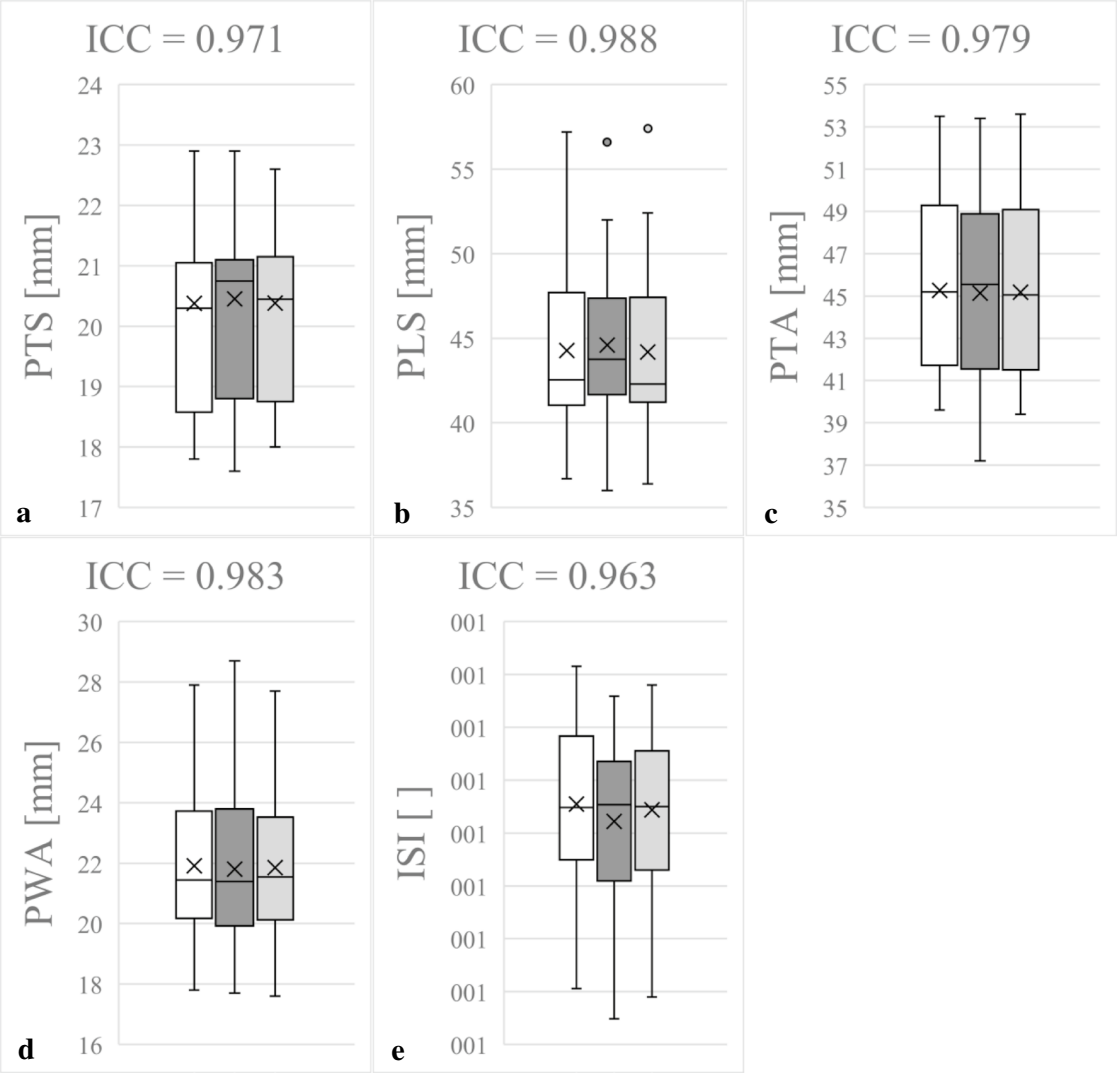
Determination of intraclass/interclass correlation coefficient (ICC) of thickness of patella in sagittal view (PTS, **a**), length of patella in sagittal view (PLS, **b**), thickness of patella in axial view (PTA, **c**), wide of patella in axial view (PWA, **d**) and Insall-Salvati-index (ISI, **e**): the white box plots represent the initial measurements by YH, the dark grey ones the second recordings by YH. The box plots marked in light grey reflect the measurements by MPM. The figures show the cumulative ICC between the initial and control measurements by YH and the observations by MPM. Outliers are marked with points. Excellent measurement reliability (ICC > 0.9) was determined for all measured parameters (PTS, PLS, PTA PWA and ISI). The control measurements were carried out at intervals of 2 weeks by the same investigator again and another one in blinded fashion based on 50 subjects

ICC for intra-observer reliability ranged from 0.81 to 0.99 and inter-observer reliability from 0.80 to 0.98, indicating results between good and excellent reliability. Taking into account the initial measurements, the control measurements by the same examiner as well as the measurements by a second examiner, cumulative ICC values of 0.81 to 0.99 (PTS 0.97, PLS 0.99, PTA 0.98, PWA 0.98, ISI 0.96) resulted. These results are summarised in Fig. 2.

### Analysis of measured patellar parameters

Table 1 shows the descriptive analysis of patellar morphology parameters in patients without radiological structural damage or radiological osteoarthritis (*n* = 409) including mean values, standard deviation, minimal and maximal measured values for PTS, PLS, PTA and PWA.

**Table 1:** Descriptive analysis of patellofemoral morphology in patients without radiological structural damage or manifested osteoarthritis (*n* = 409)

(*n* = 409)	Mean	SD	Min	Max
**PTS** [mm]	20.07	± 2.36	12.20	28.50
**PLS** [mm]	44.01	± 4.44	33.90	56.60
**PTA** [mm]	21.79	± 2.48	16.20	32.40
**PWA** [mm]	44.46	± 4.64	21.60	57.70

*SD* standard deviation, *min.* minimale, *max.* maximal

### Thickness of *patella* in sagittal view

In total (*n* = 409), a mean PTS of 20.07 mm (± 2.36 [12.20–28.50]) was found. The mean PTS in left knees was 20.24 mm (± 2.54) and in right knee joints 19.89 mm (± 2.16). For the younger patients (20–50 years, *n* = 219), a mean PTS of 19.86 mm (± 2.33) was measured, while the older patients (> 50 years, *n* = 190) showed a mean PTS of 20.32 mm (± 2.39). The analysis yielded a mean PTS of 18.95 mm (± 2.01) in female knees (*n* = 211) and of 21.27 mm (± 2.12) in male knees.

### Length of *patella* in sagittal view

Overall, a mean PLS of 44.01 mm (± 4.44 [33.90–56.60]) was found. The mean PLS in left knees was 44.37 mm (± 4.73) and in right knees 43.63 mm (± 4.11). For the younger age group, a mean PLS of 43.96 mm (± 4.50) was detected, while the older patients showed a mean PLS of 44.08 mm (± 4.40). The analysis yielded a mean PLS of 41.21 mm (± 3.04) in female knees and of 47.00 mm (± 3.72) in male knees.

### Thickness of *patella* in axial view

The analysis revealed a total mean PTA of 21.79 mm (± 2.48 [16.20–32.40]). The mean PTA in the left knees was 21.89 mm (± 2.59) and on the right joint side 21.67 mm (± 2.37). For the younger age group, a mean PTA of 21.76 mm (± 2.58) was detected. The older patients showed a mean PTA of 21.81 mm (± 2.39). The analysis yielded a mean PTA of 20.41 mm (± 1.88) in female knees and of 23.25 mm (± 2.20) in male knees.

### Width of *patella* in axial view

In total, a mean PWA of 44.46 mm (± 4.64 [21.60–57.70]) was found. The mean PWA in left knees was 44.66 mm (± 4.68) and in right knees 44.25 mm (± 4.62). For the younger age group, a mean PWA of 44.22 mm (± 4.51) was detected, while the older patients showed a mean PWA of 44.74 mm (± 4.80). The analysis yielded a mean PWA of 41.44 mm (± 3.38) in female knees and of 47.68 mm (± 3.52) in male knees.

### Analysis of side-specific differences for patellar diameters

There were no significant differences in analysis of side-specific differences of the patellar morphology parameters: PTS (*p* = 0.084), PLS (*p* = 0.115), PTA (*p* = 0.411), PWA (*p* = 0.430). All results are summarised in Table 2.

**Table 2:** Analysis of side-specific differences for patellar morphology parameters in patients with radiological healthy knee joints (*n* = 409)

	Total** (***n*** = **409**)**	Left** (n =** 212**)**	Right** (***n*** = **197**)**	*p*-value
**PTS** [mm]	20.07 (± 2.36)	20.24 (± 2.54)	19.89 (± 2.16)	0.084^1^
**PLS** [mm]	44.01 (± 4.44)	44.37 (± 4.73)	43.63 (± 4.11)	0.115^1^
**PTA** [mm]	21.79 (± 2.48)	21.89 (± 2.59)	21.67 (± 2.37)	0.411^1^
**PWA** [mm]	44.46 (± 4.64)	44.66 (± 4.68)	44.25 (± 4.62)	0.430^1^

^1^Mann-Whitney *U* test

### Analysis of age-specific differences for patellar diameters

The analysis showed significant age-specific differences for PTS (*p* = 0.031) between the two age groups (20–50 years and more than 50 years). For the younger age group, a mean PTS of 19.86 mm (± 2.33) was detected, while the older patients showed a mean PTS of 20.32 mm (± 2.39). The other patellar parameters showed no association with age. These results are summarised in Table 3.

**Table 3:** Analysis of age-specific (age in years) differences for patellar morphology parameters in patients with radiological healthy knee joints (*n* = 409)

	Total(*n*** = **409)	20–50 y.** (***n*** = **219)	** > **50 y.** (***n*** = **190**)**	*p*-value
**PTS** [mm]	20.07 (± 2.36)	19.86 (± 2.33)	20.32 (± 2.39)	0.031*^1^
**PLS** [mm]	44.01 (± 4.44)	43.96 (± 4.50)	44.08 (± 4.40)	0.640^1^
**PTA** [mm]	21.79 (± 2.48)	21.76 (± 2.58)	21.81 (± 2.39)	0.632^1^
**PWA** [mm]	44.46 (± 4.64)	44.22 (± 4.51)	44.74 (± 4.80)	0.180^1^

^1^Mann-Whitney *U* test

### Analysis of gender-specific differences for patellar diameters

Significant gender-specific differences were found for all parameters. The mean PTS in female patients was 18.95 mm (± 2.01) and 21.27 mm (± 2.12) in male patients (*p* < 0.001). Likewise, a significant (*p* < 0.001) larger PLS was found in male knee joints (47.00 mm [± 3.72]) compared to females (41.21 mm [± 3.04]). In addition, there were higher values for mean PTA and PWA (*p* < 0.001 each) of male (PTA, 23.25 mm [± 2.20]; PWA, 47.68 mm [± 3.52]) compared to female knee joints (PTA, 20.41 mm [± 1.88]; PWA, 41.44 mm [± 3.38]). These results are summarised in Table 4.

**Table 4:** Analysis of gender-specific differences for patellar morphology parameters in patients with radiological healthy knee joints (*n* = 409)

	Total (*n*** = **409)	Females (*n*** = **211)	Males (*n*** = **198)	*p*-value
**PTS** [mm]	20.07 (± 2.36)	18.95 (± 2.01)	21.27 (± 2.12)	< 0.001***^1^
**PLS** [mm]	44.01 (± 4.44)	41.21 (± 3.04)	47.00 (± 3.72)	< 0.001***^1^
**PTA** [mm]	21.79 (± 2.48)	20.41 (± 1.88)	23.25 (± 2.20)	< 0.001***^1^
**PWA** [mm]	44.46 (± 4.64)	41.44 (± 3.38)	47.68 (± 3.52)	< 0.001***^1^

^1^Mann-Whitney *U* test

### Analysis of differences between patellar diameters depending on *patella* height position

Tables 5 and 6 present the results of the ANOVA analysis and the additional post hoc test in detail. The analysis showed 65 (15.9%) patients with a patella alta, 327 (80.0%) with a patella norma and 17 (4.1%) with a patella baja.

**Table 5:** Comparison of patella morphology parameters depending on patella position after Insall-Salvati (baja, norma, alta)

	Alta (*n*** = **65)	Norma (*n*** = **327)	Baja (*n*** =** 17)	*p*-value
**PTS** [mm]	18.87 (± 2.15)	20.22 (± 2.32)	21.85 (± 2.08)	< 0.001***^1^
**PLS** [mm]	40.56 (± 3.11)	44.41 (± 4.19)	49.64 (± 4.54)	< 0.001***^1^
**PTA** [mm]	20.82 (± 2.03)	21.88 (± 2.49)	23.65 (± 2.38)	< 0.001***^1^
**PWA** [mm]	42.21 (± 3.73)	44.80 (± 4.70)	46.56 (± 3.92)	< 0.001***^1^

^1^Ordinary one-way ANOVA

**Table 6:** Post hoc analysis of ordinary one-way ANOVA (Table 5) based on comparison of patella morphology parameters depending on patella position after Insall-Salvati

		Alta/norma	Alta/baja	Norma/baja
**PTS** [*p*-value]		< 0.001***^1^	< 0.001***^1^	0.013*^1^
**PLS** [*p*-value]		< 0.001***^1^	< 0.001***^1^	< 0.001***^1^
**PTA** [*p*-value]		0.004**^1^	< 0.001***^1^	0.010*^1^
**PWA** [*p*-value]		< 0.001***^1^	0.001**^1^	0.267^1^

^1^Tukey’s multiple comparison test

Significant differences (*p* < 0.001) were detected for all patellar diameters: PTS (alta, 18.87 mm [± 2.15]; norma, 20.22 mm [± 2.32]; baja, 21.85 mm [± 2.08]), PLS (alta, 40.56 mm [± 3.11]; norma, 44.41 mm [± 4.19]; baja, 49.64 mm [± 4.54]), PTA (alta, 20.82 mm [± 2.03]; norma, 21.88 mm [± 2.49]; baja, 23.65 mm [± 2.38]) and PWA (alta, 42.21 mm [± 3.73]; norma, 44.80 mm [± 4.70]; baja, 46.56 mm [± 3.92]).

The post hoc analysis between alta/norma, alta/baja and norma/baja revealed in all examination cases significant differences for the parameters mentioned above, with the exception of PWA in the comparison of patella norma and baja (Table 6).

Moreover, a gender-specific difference was found for the ISI (*p* < 0.001). Men had a mean ISI of 1.02 (± 0.15) and women of 1.07 (± 0.16).

## Discussion

The etiology of AKP is often uncertain, both in young athletic patients and in older patients after endoprosthetic joint replacement, despite extensive diagnostics [6–10, 14–16]. Some studies indicate that pathological patellar height positions can result in AKP [17–20]. Physiologic ISI–dependent radiologic reference values of the patellofemoral morphology are required to improve the diagnostics and therapy of uncertain knee pain in knee joint preserving and replacing surgery. To this purpose, 409 MRI scans of adult knee joints were included in the presented study to investigate side-, age- and gender-specific differences in the morphology of the patellofemoral joint and additionally to decipher potential dependencies on patellar height position.

Using a CT scan of 37 cadavers from different time periods, Monk et al. [31] demonstrated that the morphology of the patellofemoral joint has changed continuously over centuries. The authors concluded that the morphological changes in the shape of sulcus flattening and lateralization of the patella could have resulted in AKP [31].

Many comparative studies present reference values for the morphology of the distal femur, in particular the trochlea [32–34]. However, there are only a few studies of patellar morphology in a population of patients with low grade or absent osteoarthritis in the current literature [35, 36]. Therefore, the measured values presented here provide important references for a radiological “healthy” population.

In the present study, no side-specific differences in the morphology of the patella could be demonstrated. This finding is consistent with the current literature [37, 38]. For example, Dagneaux et al. [37] observed no significant differences in the morphology of the patella in a CT examination of 345 patients comparing both knee joints.

The described results showed an increase in PTS with raising age. These changes can be explained by incipient osteoarthritic changes. Degeneration of the retropatellar cartilage changes the pressure conditions on the subchondral bone. This could possibly lead to an increase in the thickness of the subchondral bone. These assumptions are supported by current comparative studies [39–41].

The analysis of the presented study revealed significantly higher measured values for all patellar measurement parameters in the male knee joints compared to the female knee joints. The average larger dimensions of the male knee joint compared to the female knee joint were also found in other comparative studies [42–44]. The results of the present studies represent important gender-specific reference values for the patellar diameters. The findings provide relevant guide data for reconstruction of the physiological morphology of the patellofemoral joint, especially during implantation of an endoprosthetic retropatellar replacement.

Interestingly, the presented study establishes a relationship between the patellar height position and the patellar diameter for the first time. Knee joints with a patella baja showed larger mean diameters than knee joints with a patella alta. In addition, the patellar diameters also differed in comparison to the patella norma. The present results suggest that the patellar diameters show smaller measured values on average with a larger ISI (patella baja > patella norma > patella alta).

One possible explanation for these findings could be a relation to the retropatellar contact pressure. In a simulator study, Luyckx et al. [17] were able to demonstrate that a patella alta increases the intraarticular patellar contact pressure. This conclusion was confirmed by Watson et. al [45] in a finite element model. The smaller patellar diameters of patients with a patella alta in the present radiologically primary asymptomatic collective could be a physiological compensation mechanism to reduce the patellar contact pressure, which are caused by the higher patella position in the presence of patella alta. Due to the observational study design, the present results cannot sufficiently prove this hypothesis. Therefore, prospective clinical studies are needed to verify this hypothesis. The illustrated results should be taken into account as an important reference when conducting such studies.

In the gender-specific analysis of the ISI, a significantly smaller ISI was found in men (1.02) than in women (1.07). Even if the mean values are within the definition range of the patella norma in each case, the known average greater extent of the patellar diameters in men could provide an explanation for the presented results if it is assumed that men tend to have a patella baja on average and women tend to have a patella alta.

However, it should be noted that the reference values used for the ISI are based on X-ray images. Shabshin et al. [46] examined 262 MRIs of knee joints to define MRI-based reference parameters for the ISI. The authors argued for a new reference interval with the range from 0.74 to 1.5 [46]. According to these criteria, a patella baja would have been detected in only one investigation in the presented collective and the remaining examinations would have been classified as patella norma. Regardless of the nomenclature, it can be stated that a lower patellar height is associated with larger patellar diameters and that on average men appear to have a smaller ISI than women.

A limitation of the study is that there was no comparison of the MRI scans with related CT imaging, because many of the included patients were only examined via MRI, but not via CT. A comparison between the different modalities seems useful. The present study could thereby have made an even better comparison with the results of other authors. In general, MRI is inferior to CT in terms of imaging osseous structures. The measurements performed could therefore not always be carried out in the best possible section plane.

Furthermore, a comparison of the Caton-Deschamps index and the results of the ISI would have been interesting. An intermodal comparison between radiograph, CT and MR imaging in the investigation of the Caton-Deschamps index and ISI would be desirable for future studies.

As a last point, it should be noted that the reported findings are purely descriptive in nature. A statement about the clinical occurrence of an AKP cannot be made on the basis of the presented data.

In summary, the results showed an increased PTS with rising patient age, which could be a reaction of the subchondral retropatellar bone as a consequence due to degeneration of the patellofemoral joint. In accordance with the current literature, gender-specific differences in the patellar diameters were found. All measured parameters were higher in male knee joints in comparison to the female knee joint. Moreover, relationships between the patellar height position and the patellar diameters were found. In general, a smaller ISI seems to be associated with bigger patellar diameters. This insight should be investigated in further studies and taken into account in everyday clinical practice as a radiological reference in the diagnostics and therapy of knee joint preserving and replacing surgery.

## Data Availability

All data generated or analysed during this study are included in this published article.

## Abbreviations

AKP: Anterior knee pain
CT: Computed tomography
ICC: Intraclass correlation coefficient
ISI: Insall-Salvati index
KL: Kellgren/Lawrence
MRI: Magnetic resonance imaging
PLS: Length of patella in sagittal view
PTA: Thickness of patella in axial view
PTS: Thickness of patella in sagittal view
PWA: Wide of patella in axial view
